# Protein biomarkers distinguish between high- and low-risk pediatric acute lymphoblastic leukemia in a tissue specific manner

**DOI:** 10.1186/1756-8722-6-52

**Published:** 2013-07-12

**Authors:** Maria Braoudaki, George I Lambrou, Konstantinos Vougas, Kalliopi Karamolegou, George T Tsangaris, Fotini Tzortzatou-Stathopoulou

**Affiliations:** 1 First Department of Pediatrics, Choremeio Research Laboratory, University of Athens Medical School, Thivon & Levadias, Goudi-Athens 11527, Greece; 2 Proteomics Research Unit, Center of Basic Research II, Biomedical Research Foundation of the Academy of Athens, Athens, Greece; 3 First Department of Pediatrics, University of Athens Medical School, Choremeio Research Laboratory, Thivon & Levadias 11527 Goudi-Athens, Greece

**Keywords:** Childhood leukemia, Mass spectrometry, Proteomics, Two-dimensional gel electrophoresis

## Abstract

The current study evaluated the differential expression detected in the proteomic profiles of low risk- and high risk- ALL pediatric patients to characterize candidate biomarkers related to diagnosis, prognosis and patient targeted therapy. Bone marrow and peripheral blood plasma and cell lysates samples were obtained from pediatric patients with low- (LR) and high-risk (HR) ALL at diagnosis. As controls, non-leukemic pediatric patients were studied. Cytogenetic analysis was carried out by G- banding and interphase fluorescent *in situ* hybridization. Differential proteomic analysis was performed using two-dimensional gel electrophoresis and protein identification by matrix-assisted laser desorption ionization time-of-flight mass spectrometry. The differential expression of certain proteins was confirmed by Western blot analysis. The obtained data revealed that CLUS, CERU, APOE, APOA4, APOA1, GELS, S10A9, AMBP, ACTB, CATA and AFAM proteins play a significant role in leukemia prognosis, potentially serving as distinctive biomarkers for leukemia aggressiveness, or as suppressor proteins in HR-ALL cases. In addition, vitronectin and plasminogen probably contributed to leukemogenesis, whilst bicaudal D-related protein 1 could afford a significant biomarker for pediatric ALL therapeutics.

## Introduction

Pediatric acute lymphoblastic leukemia (ALL) is a malignancy that accounts for an approximate 25 to 35% of all cancer cases among children and is generally favorable, with cure rates presently exceeding 80% [1,2]. In most treatment protocols, different genetic subtypes of childhood ALL are treated following risk-adapted therapy, which is tailored to the patients’ relative risk of relapse [3]. The evaluation of the risk of relapse is essential at diagnosis to prevent under- or over-treatment [4]. Current risk stratification concerning ALL, is based on certain criteria including early response to therapy, clinical, biological and pharmacogenetic features, such as the patient’s age and white blood cell count (WBC) at diagnosis, as well as the genetic characteristics of leukemic cells [5,6]. The accurate assignment of patients to different risk groups is vital to decide the premium therapeutic strategy for each case [7].

Hitherto, despite incremental therapeutic improvements, significant subsets of children systematically relapse, carrying a dismal prognosis and therefore highlighting the need to further increase the survival rate and improve the quality of life for pediatric patients with ALL. Hence, effective target identification therapies represent the most critical clinical approach for children with high-risk ALL. However, to design individualized therapy, knowledge of the biology of the malignancy and the host is essential for an improved therapeutic outcome [2,3].

Ongoing research has attempted to identify novel targets for therapeutic interventions, which will increase the efficacy of current treatments, enable the development of personalized ALL therapy and subsequently improve patients’ clinical outcomes [8-16]. Comparative proteomic profiling is considered a promising practical approach with broad application in clinical biological science [13]. Proteomics allow the examination of expression profiles at the protein level on genome-wide scale, providing insights into new diagnostic and therapeutic targets [17]. Additionally it allows the identification of potential biomarkers, which might be predictive for the use of a more targeted approach in treatment [18].

In the current setting, data is presented on changes in protein expression levels in the bone marrow (BM) and peripheral blood (PB) plasma and cell lysates of pediatric patients diagnosed with low- and high-risk ALL, to identify novel biomarkers related to diagnosis, prognosis and in particular, patient tailored therapy.

## Materials and methods

### Patients & samples

Bone marrow and PB samples were analyzed from 45 pediatric patients with B-ALL. In total, 39 patients had common ALL, 2 patients were found with pre-B and 3 with pro-B, whereas in one case mature L3-ALL was diagnosed. The diagnosis of ALL was based on French-American-British (FAB) Cooperative Group criteria and immunophenotype scheme [19]. The patient population comprised primarily of low middle class (*n* = 44), Greek Orthodox children (*n* = 45) coming from the geographical region of Continental Greece. The patients’ median age was 4.07 ± 4.11 yrs (*n* = 45) and among them, 21/45 (46.7%) children were males. Females’ median age was 4.09 ± 3.48 yrs (*n* = 24) and males’ median age was 2.92 ± 4.70 yrs (*n* = 21). Patient data are also presented as Supplementary file (Additional file 1: Figure S1). Patients were assigned as low risk (LR) when aged from 1 to 9 years old, with a WBC count < 50x10^9^/L and L2 < 20% without CNS involvement. High risk (HR) patients were considered those aged <1 or >10 years old, with WBC count > 50x10^9^/L, L2 > 20% or L3 blasts and CNS disease. All patients received chemotherapy according to the modified HOPDA-97 protocol [20]. Among them, 37 patients (82%) entered complete morphological or clinical remission (CR) and remain alive up to date. Eight patients succumbed (18%). Of note, 6/8 patients remained in CR before decease. In general, patients succumbed following relapse 3 years after initial diagnosis (2/45; 4.4%), infections (3/45; 6.7%), another cause (2/45; 4.4%) and Crohn’s disease (1/45; 2.2%). Studied and used as controls, were BM and PB samples from seven non-leukemic pediatric patients. All BM and PB specimens were collected before initiation of cytotoxic therapy. The isolation of BM and PB plasma (BMP and PBP, respectively) and BM and PB cell lysates (BMC and PBC, respectively) was performed as previously described [21]. All samples were stored at −80°C until used and protein concentrations were determined by Bradford Reagent (Bio-Rad, Hercules, CA, USA). The study was conducted with the approval of the ethics committee of the Medical School of the University of Athens in Greece.

### Cytogenetics

Cytogenetic investigations were performed by G-banding analysis in all patients at diagnosis. Additionally, interphase fluorescence *in situ* hybridization (iFISH) [5,22] was used to monitor *TEL/AML1* fusion gene t(12;21)(p12q22), *BCR/ABL* fusion gene t(9;22)(p34q11), *PBX1/E2A* fusion gene t(1;19)(q23p23) and mixed lineage leukaemia *(MLL)* gene rearrangements t(4;11) (q21q23).

### Protein depletion

Pre-fractionation of high abundant proteins was performed in plasma isolated from BM specimens. They derived from all three groups analysed, using ProteoMiner protein enrichment (Biorad, Hercules, CA, USA) and Vivapure Anti-HSA kits (Sartorius Stedium Biotech, Gottingen, Germany); both following manufacturer’s recommendations.

### Two-dimensional electrophoresis

Two-dimensional gel electrophoresis (2DE) was performed as previously described [23]. In brief, protein was cup-loaded and isoelectric focused on an IPGphor isoelectric system. Second-dimension electrophoresis was performed in 12% SDS-polyacrylamide gels using PROTEAN apparatus (Bio-Rad Hercules, CA, USA). The gels were stained with colloidal Coomassie Blue G250 (Novex, San Diego, CA, USA) and scanned in a GS-800 Calibrated Densitometer (Bio-Rad, Hercules, CA, USA). Spot detection, quantification and alignment, were performed using the PD-Quest v8.0 2DE analysis software. All samples were run (for) at least two times to determine variability and each on several gels with different pH range, including 3-10NL and 4-7L.

### Peptide mass fingerprinting

All spots were excised by the Proteiner SPII (Bruker Daltonics, Bremen, Germany) and dried in a speed vacuum concentrator (MaxiDry Plus, Heto, Denmark). The MS analyses were performed on mass spectra of matrix-assisted laser desorption/ionization-time-of-flight-mass spectrometry (MALDI-TOF-MS) (Ultraflex II, Bruker Daltonics, Germany). The detailed procedure is described by Kollialexi et al. [24].

### Protein interaction network analysis

Differentially expressed proteins, identified in the present study, were used for pathway analysis. For this purpose, the Swiss-Prot accession numbers were inserted into the STRING (Search Tool for the Retrieval of Interacting Genes/Proteins’) software, which is available at http://string.embl.de/ [25].

### Western blot

Ceruloplasmin, clusterin and apolipoprotein A1 antigens were detected using primary monoclonal antibodies (sc69767, sc56079, sc58230, respectively; Santa-Cruz Biotechnology Inc. CA, U.S.A.) at a dilution of 1:200 overnight at 4°C, as previously described (Braoudaki et al., 2010a). The corresponding anti-mouse HRP-conjugated secondary antibody (Santa-Cruz Biotecnology Inc. CA, U.S.A.) was added at a dilution of 1:5000. The obtained signals were compared to IgG (sc69786; Santa-Cruz Biotechnology Inc., CA, USA) as internal standard. All bands were visualized using the enhanced chemiluminescence (ECL *west pico*) detection system (Pierce Biotechnology Inc., Rockford, U.S.A.). Western blots were scanned with a GS-800 Calibrated Densitometer (Bio-Rad, Hercules, CA, U.S.A.) and images were analyzed by Quantity One image processing software (Bio-Rad, Hercules, CA, USA. All experiments were carried out in triplicate.

### Data analysis and statistical evaluation

Mean densitometry values of all individual protein spots were obtained from each sample using *PDQuest*® image processing software (Bio-Rad, Hercules, CA, USA). Protein intensity values were obtained by calculating the mean of each protein detected in every patient group. Densitometry levels were first evaluated by the one-sample Kolmogorov-Smirnov Goodness-of-Fit test, in order to determine whether they followed a normal distribution pattern. The non-parametric Spearman rank correlation was used to examine pair-wise correlations between different protein levels and their association with continuous variables (age, WBC count, diagnosis etc.). T-test was used to study differential protein expression, as compared to control samples. One-way ANOVA, n-way ANOVA and Kruskal-Wallis tests have been used to examine the expression status of the proteins with various clinicopathological parameters before and after stratification. Actuarial estimates of the leukemia free survival (LFS) and the overall survival (OS) were estimated using the Kaplan-Meier method. More specifically, it was used to estimate LFS and OS as functions of time along with Log-rank (Mantel Cox) and Gehan-Breslow-Wilcoxon tests. Numerical values are expressed as the mean ± standard deviation (SD). Values were considered significant when p < 0.05. OS denotes the percentage of patients that survived for a certain period of time since diagnosis or treatment completion. LFS was calculated from the date of diagnosis to date of leukemic transformation (uncensored) or last contact/date of death (censored).

Protein data classification was performed using Hierarchical Clustering (HCL) and Principal Component Analysis (PCA) [26]. In order to compare different groups of proteins, highlighting different functionalities among all experimental setups, interesting proteins formed study sets. These were further subjected to Gene Ontology (GO) based analysis to test the nature of the underlying risk mechanism. The chosen approach was the parent–child-union method [27], since it was found to outperform the standard method of overrepresentation analysis (ORA) in GO. The standard approach treats each GO term independently and hence does not take dependencies between parent and child terms into account, ignoring the structure of the GO hierarchy. It was shown that this behavior can result in certain types of false positive results, with potentially misleading biological interpretation [27]. In contrast, the parent–child method measures the overrepresentation of a term with respect to the presence of its parental terms in the set. Hence, it resolves the problem of the standard method, which tends to falsely detect an overrepresentation of more specific terms below of terms known to be overrepresented.

ORA was performed with the publicly available Ontologizer 2.0 tool [27,28] using GO terms definitions and associations between proteins and GO downloaded from the Gene Ontology consortium [29] on the 26th of November 2010. Also, GO analysis was performed using the *WebGestalt* web-tool [30] as an alternative method.

The differentially expressed proteins were mapped on different pathways using the *Pathway Explorer* software (*Technische Universitaet-Graz, Austria*) [31]. First of all, the percentage of proteins present in all known pathways was investigated using the databases available through the *Pathway Explorer* software. Alternatively, the *WebGestalt* web-tool [30] was used for pathway analysis. All analyses have been performed with the *MATLAB* Computing environment (The Mathworks, Inc. Natick, MA).

## Results

### Patients characteristics

Patient clinical data and demographics are summarized in Table 1. To establish protein expression profiles, the pediatric patients were assigned to certain risk groups. Hence, the series comprised of 19 (42.2%) LR- and 26 (57.8%) HR-ALL patients (Table 1).

**Table 1:** Summary of clinical data of patients used in the present study

**Inv.**	**Assigned code**	**Gender**	**Diagnosis**	**Age at diagnosis (yrs)**	**Survival**	**Survival time (yrs)**	**(WBC) [x10**^**3**^**/ul]**	**TEL/AML1**	**BCR/ABL**	**MLL**	**Karyotype**	**RISK**	**MRD**	**Clinical outcome**
1	X1	F	common	2.43	1	2.93	1200.00	-	-	-		L	-	CR
2	X2	M	common	4.07	1	2.75	3570.00	-	-	-	46XY	L	-	CR
3	X3	F	common	4.10	1	3.15	5700.00	-	+	-	46ΧΧ, one clone with chromosome 7 t(1;19)	H	-	CR
4	X4	M	common	2.75	2	1.12	48000.00	+	-	-	46ΧΥ, ΤδΤ-	H	-	CR
5	X5	M	common	7.77	1	3.01	18800.00	-	-	-	46XY	H	-	CR
6	X6	M	pro-B	2.35	1	6.47	4400.00	-	-	-	trisomy 4, 6,18,22. X trisome in 5 metaphases	H	-	CR
7	X7	M	common	5.13	1	4.36	13800.00	-	-	-		L	-	CR
8	X8	M	common	2.19	2	0.45	7100.00	-	-	-		H	-	CR
9	X9	M	common	5.28	1	0.63	8050.00	-	-	-		L	-	CR
10	X10	M	common	1.83	2	3.84	58600.00	-	-	-	50ΧΥ, , +Χ, t(5;7)(p11;q11), +14,add(14)(p11.1)+21,+21[13]/46,XY[1], hyperdiploidy: trisomy 14, 21, 22, Χ, total chromosomes 50, in one metaphase, 51 chromosomes with tetrasome 21	H	-	Relapse
11	X11	F	pre-B	4.08	1	2.54	1220.00	-	-	-		L	-	CR
12	X12	F	common	6.72	1	4.05	67980.00	-	-	-	46ΧΧ	H	-	CR
13	X13	F	common	2.94	1	3.79		+	-	-	46ΧΧ	L	-	CR
14	X14	F	common	1.01	1	3.32	42000.00	-	-	-		H	-	CR
15	X15	F	common	5.80	1	4.99	4370.00	-	-	-	56XX, trisomy 4,6,8,10,11,14,15, tetrasomy 21,	H	-	CR
16	X16	F	common	6.05	2	1.82	137000.00	-	+	-	46XX, t(9;22)(q34;q1) mutual translocation between chromosomes 9 +1, chromosome 22, BCR/ABL: 92%	H	-	CR
17	X17	M	pre-B	14.85	2	1.54	92960.00	-	+	-	45ΧΥ, BCR/ABL 97,2%+ MLL- TEL/AML1-	H	-	CR
18	X18	M	common	1.93	1	4.76	5600.00	-	-	-	59XXY, hyperdiploidy +4,+6,+8,+29,+10,+11,+15,+17,+20,+21*2,	H	-	CR
19	X19	M	common	2.02	1	5.01	1380.00	-	-	-	46XY	L	-	CR
20	X20	F	common	2.23	1	2.11	9670.00	-	-	-		L	-	CR
21	X21	F	common	2.86	1	4.50	14700.00	-	-	-		L	-	CR
22	X22	F	common	3.30	1	2.67	30820.00	+	-	-		L	-	CR
23	X23	M	common		1	2.56		-	-	-		H	-	CR
24	X24	M	common	14.04	2	3.11	670.00	-	-	-		H	-	Relapse
25	X25	F	common	4.41	1	5.00	10440.00	-	-	-		L	-	CR
26	X26	F	common	13.72	1	3.09	4000.00	+	-	-	46XX, t(1;19)(q23p13)83,7%	H	-	CR
27	X27	F	common	14.41	1	4.56	2220.00	-	-	-	55XX+X, +4,+8, +14, +17, +18, +21 [8]/46XX[2]	H	-	CR
28	X28	M	common	2.92	1	2.09	8100.00	-	-	-		L	-	CR
29	X29	F	common	5.25	1	4.37	6520.00	+	-	-	46ΧΧ	L	-	CR
30	X30	F	L3	5.49	1	4.03	3700.00	-	-	-	49XX, xx,+4, +21,/46XX	H	-	CR
31	X31	F	common	-0.70	1	3.86	74800.00	-	-	+	46XX	H	-	CR
32	X32	F	common	4.97	2	1.42	10610.00	-	-	-	46XX	L	-	CR
33	X33	F	common	6.97	1	4.11	4000.00	-	-	-		H	-	CR
34	X34	M	common	7.86	1	4.02	10270.00	+	-	-	46 XY	L	-	CR
35	X35	F	pro-B	3.23	1	3.46	22900.00	+	-	-	46xx, , TEL/AML1: 97.3%, E2A'+	L	-	CR
36	X36	M	common	2.87	1	4.15	6670.00	+	-	-		L	-	CR
37	X37	M	common	14.21	1	2.75	21000.00	-	+	-	46XY, 1(q)(q0) del13 del19 t(1;19)-del13, BCR/ABL 90,7%+ TCF3E2A/PBX1 (1;19) 95%+	H	-	CR
38	X38	M	common	7.47	1	3.50	55050.00	-	-	-		H	-	CR
39	X39	M	common	13.61	1	7.75		-	-	-		H	-	CR
40	X40	F	common	3.13	1	4.08	17000.00	-	-	-		L	-	CR
41	X41	M	common	1.75	1	2.93	4400.00	+	-	-	Tcf3 (E2A)-, AML1: 4 copies (+)50%	L	+	CR
42	X42	F	pro-B	9.31	1	3.04	3000.00	+	-	-	TEL/AML(78,7%)	H	-	CR
43	X43	M	common	1.01	1	3.57	8800.00	-	-	+	48XY+X mutual translocation 11 or 19. 1 extra Χ, translocation between 2 Η 9, 1 extra chromosome 6, MLL 92%	H	-	CR
44	X44	F	common	0.16	1	3.84	84000.00	-	-	+	46XX, MLL:95.8%,	H	+	CR
45	X45	F	common	3.22	2	2.52	33280.00	+	-	-	46ΧΧ/49ΧΧ,+Χ, +10 +21 ΤΕL/AML96,6%	H	-	CR

(Legends: *Inv*: Invoice, *Assigned Code*: Internal patient laboratory code,*Survival*: *1*: Alive, *2*: Deceased, *MRD*: Minimal Residual Disease) MRD detection at the end of induction (28^th^ day), *Risk*: *L*:Low, *H*:High.

### Protein analyses

From each ALL patient, including LR- and HR-ALL, BM and PB plasma and BM and PB cell lysates were electrophoresed (total 4 gels/patient). All samples were run on gels with 3-10NL and 4-7L pH ranges. Bone marrow and PB plasma samples were depleted of high abundant proteins using ProteoMiner and Vivapure Technologies independently and were run on gels with 3-10NL pH ranges. The overall number of gels evaluated in the relevant groups was 468. Accordingly, a total of 84 samples were analyzed in seven non-leukemic patients (12 samples/patient), which served as controls.

### Protein identification in BM plasma samples

**Figure 1:**
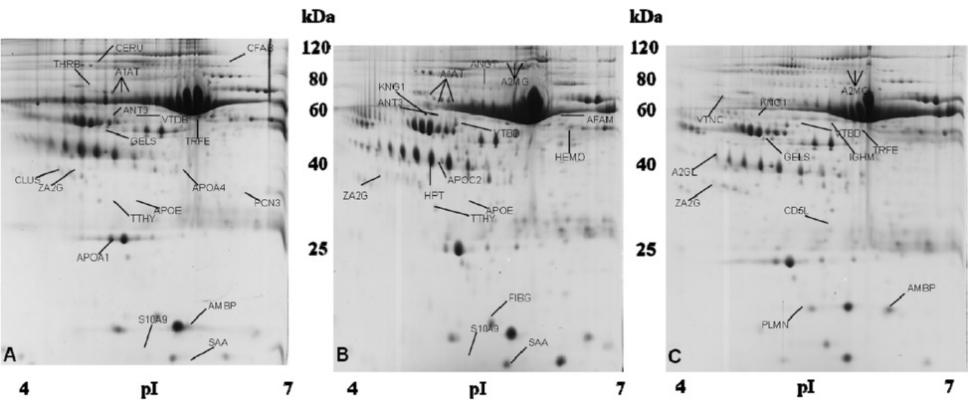
**Representative gel images of BM plasma derived from LR- (A), HR-ALL (B) and non-leukemic (C) patients.** The differentially expressed spots are annotated and indicated by arrows.

A mean of 361 ± 22 spots per gel were compared between BM plasma samples and controls and in total 46 proteins were found to be differentially expressed; 18 proteins in HR-ALL patients and 16 in LR-ALL patients (Figure 1). Among them, there were six proteins worth mentioning [ceruloplasmin (CERU), clusterin (CLUS), prothrombin (THRB), alpha-1-microglobulin/bikunin precursor (AMBP), vitamin D-binding protein (VTDB) and ficolin-3 (FCN3)], which were found up-regulated and a further two gelsolin (GELS) and protein S100-A9 (S10A9) found down-regulated in BM plasma derived from HR-ALL patients. Regarding the most considerable proteins identified in BM plasma from LR-patients, VTDB and kininogen-1 (KNG1) were found overexpressed, whilst S10A9 and afamin (AFAM) were significantly down-regulated. Importantly, KNG1 was found significantly up-regulated in the LR-ALL group of patients when compared to the HR-ALL. Overall, twelve proteins were found differentially expressed in ALL patients, independently of the risk them, GELS, KNG1, CD5 antigen (CD5L), leucine-rich alpha-2-glycoprotein precursor (A2GL), vitronectin (VTNC) and Ig mu chain C region (IGHM) were down-regulated, whereas increased expression of ZA2G, VTDB, TRFE, plasminogen (PLMN), alpha-2-macroglobulin (A2MG) and AMBP was detected. The expression level of all these proteins was altered significantly (Additional file 2: Table S1).

It is notable that significantly increased levels of AMBP and VTDB were observed in both LR- and HR-ALL patients when compared to the controls. In addition, decreased level of expression of GELS was observed in all samples from HR-patients compared to control group. A subset of proteins, that were found differentially in BM plasma samples, derived from both LR- and HR-ALL patients when compared to the BM controls were acute phase proteins including serum amyloid A (SAA) and A2GL. Of note, the levels of this group of proteins fluctuate in response to infection or injury.

### Protein identification in PB plasma samples

Regarding the proteins extracted from PB plasma samples, 25 proteins were found differentially expressed from the 401 ± 21 spots per analyzed gel, between the PB samples derived from both patients’ risk groups compared to the controls. The majority of differentially expressed proteins (89.2%) were common between LR- and HR-ALL patients, with the exceptions of AFAM. Decreased levels of AFAM were observed only in LR-ALL patients as well as AMBP and GELS, which were found merely up- and down-regulated in HR-ALL patients, respectively when compared to the control groups. In addition, it is notable that KNG1 was significantly up-regulated in the LR-ALL group of patients, compared to the HR-ALL group. Interestingly, most detected proteins in the PB samples were acute phase proteins, metabolic enzymes, structural proteins, signal transduction mediators and immunoglobulins (Additional file 2: Table S2).

### Protein identification in BM and PB cell lysates

Overall, 15 proteins were found differentially expressed between BM cell lysates and control groups (899 ± 40 spots analyzed per gel BMC/ALL sample identity), whilst 13 proteins were altered in the PB cell lysates compared to the control (890 ± 51 spots tested per gel PBC/ALL sample identity). No disparities were observed regarding protein identification between LR- and HR-ALL patients. It is noteworthy that the metabolic enzyme catalase (CATA) was detected only in PB cell lysates; previously unidentified when screening BM and PB plasma samples (Additional file 2: Table S3). Functional analysis revealed primarily the presence of acute phase proteins, metabolic enzymes and signal transduction mediators.

### Protein identification following depletion in BM plasma samples

**Figure 2:**
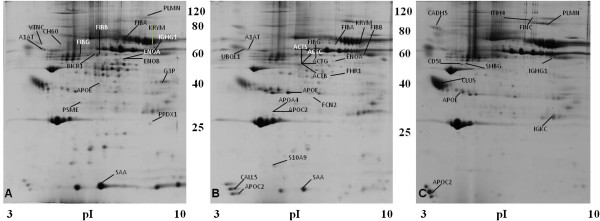
**Representative gel images of pre-fractionated BM plasma samples derived LR- (A), HR-ALL (B) and non-leukemic (C) patients.** The differentially expressed spots are annotated and indicated by arrows.

Following protein depletion, 48 proteins were found differentially expressed from the 426 ± 37 spots analyzed per gel; 20 proteins in HR-ALL, 17 proteins in LR-ALL and 11 proteins in the control group (Figure 2). Among them, examples of low abundant proteins found up-regulated in BM samples derived from HR-ALL were FCN3, calmodulin-like protein 5 (CALL5) and pyruvate kinase isoenzymes M1/M2 (KRYM), whereas ubiquillin 1 (UBQL1) was found to be down-regulated. Enhanced levels of expression were observed in LR-ALL for bicaudal D-related protein 1 (BICR1), proteasome activator complex subunit 1 (PSME 1), heat shock protein 60, (CH60), peroxiredoxin 1 (PRDX1) and KRYM when compared to control groups, among others. The latest was also up-regulated in HR-ALL compared to the control group. Of note, sex-hormone binding globulin (SHBG) and fibronectin (FINC) were detected only in the control group compared to both LR- and HR-ALL, suggesting the decreased expression of both proteins in ALL-patients, irrespectively of the risk group (Additional file 2: Table S4).

### Protein identification with respect to cytogenetics

In this study, chromosomal aberrations were observed in 24/45 (53%) cases. In 11/45 (24.4%) patients the *TEL/AML1* fusion gene t(12;21) (q21;q22) was detected, whilst in 4/45 (8.8%) cases extra *AML1* signals were identified, without *TEL/AML1* fusion. Of note, one of the *TEL/AML1* positive patients succumbed following *Pneumocystis carinii* pneumonia. The high-risk genetic aberrations such as mixed lineage leukemia *(MLL)* gene rearrangements with chromosome 11q23 abnormality and the Philadelphia chromosome *(BCR/ABL)* were detected in 3 infants (3/45; 6.7%) and in 4/45 (8.8%) cases, respectively. One of the patients carrying t(9;22)(p34q11) succumbed after fungal infection. In addition, the *PBX1/E2A* fusion gene was identified in 2/45 cases (4.4%). Overall, no association was documented between protein expression levels in patients bearing the described cytogenetic abnormalities with the patients that did not.

### Potential protein discrimination between risk assessment and karyotype

**Figure 3:**
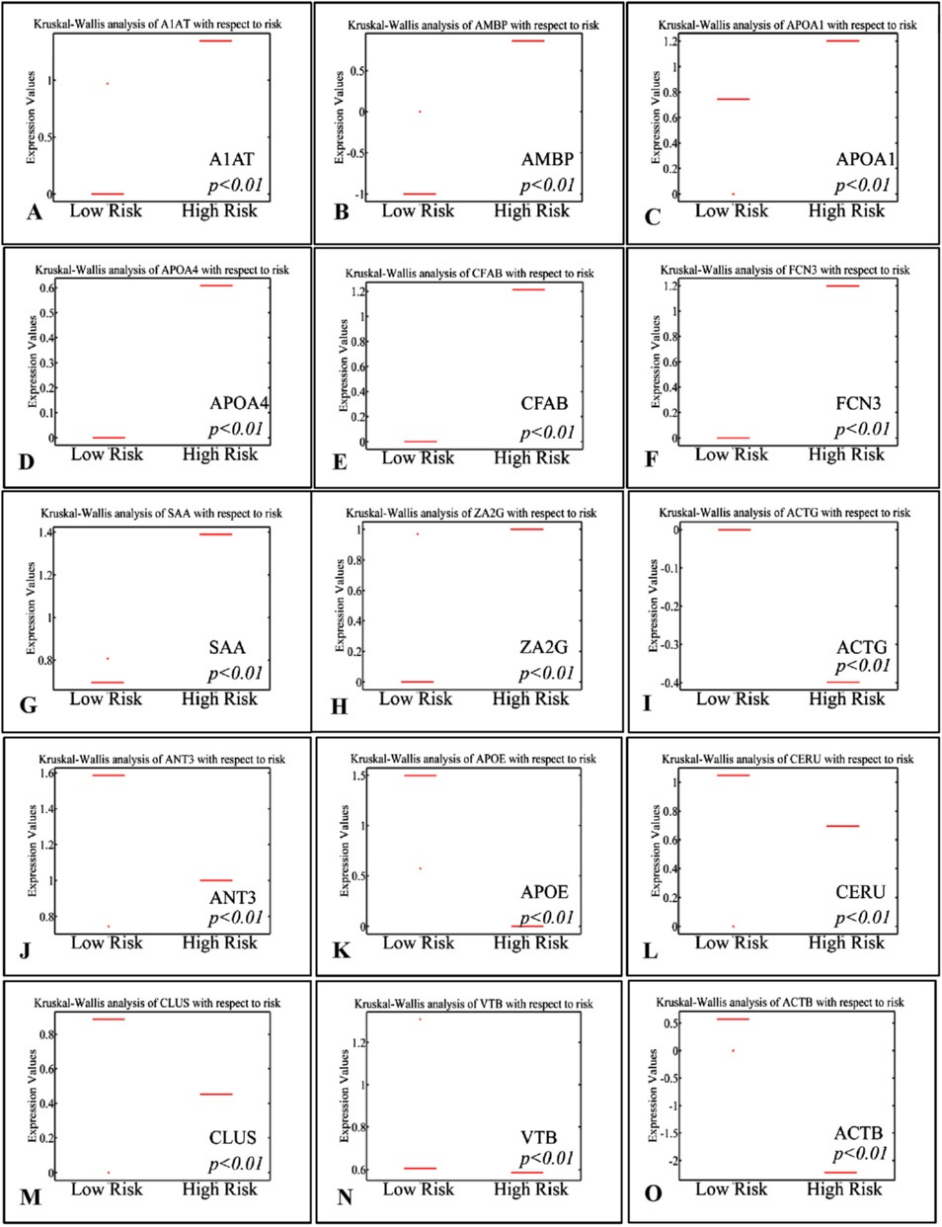
**Kruskal-Wallis analysis of protein levels with respect to risk stratification.** Up-regulation in high risk **(A-H)** and down-regulation in high risk **(I-O)**.

**Figure 4:**
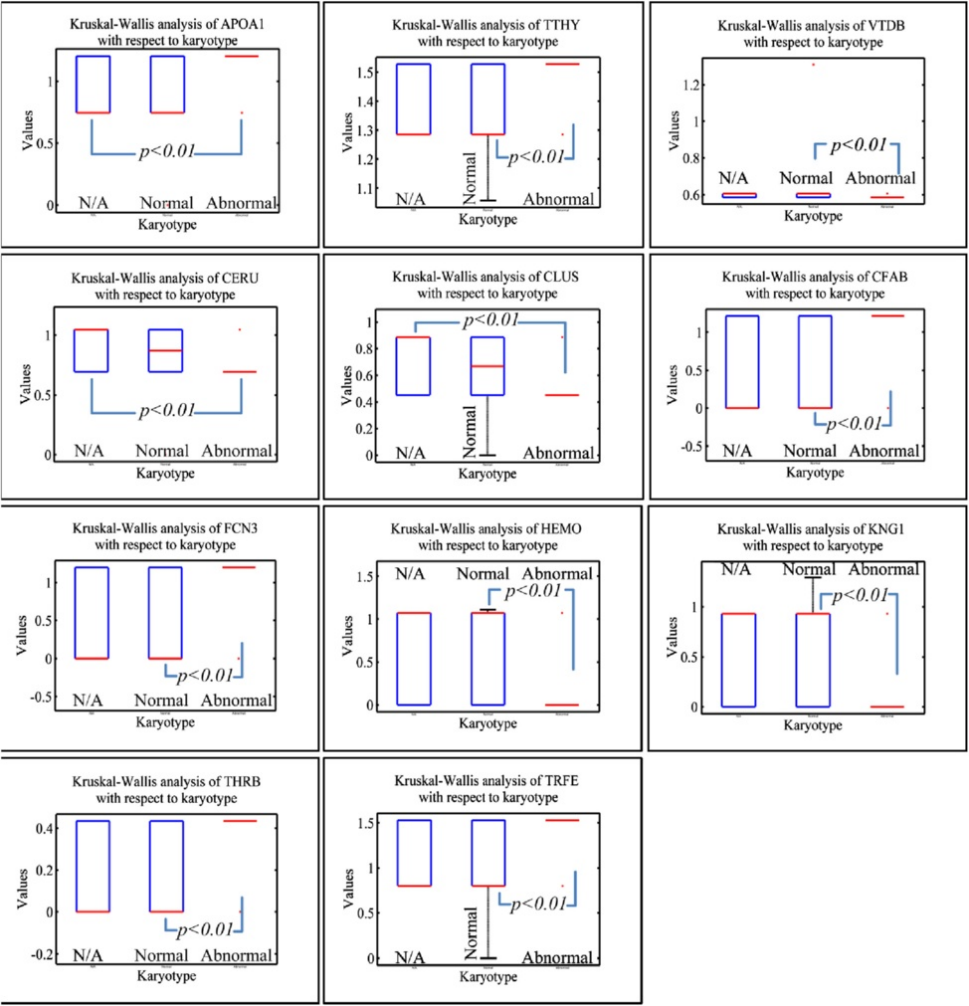
Kruskal-Wallis analysis of protein expression with respect to karyotype.

A summary of protein expression data was created (Additional file 2: Tables S1, S2, S3 and S4) in such a format that would be useful for further processing; hierarchical clustering, k-means clustering and principal component analysis (PCA) (Table 2). Protein expression was analyzed according to risk assessment and karyotype. It appeared that certain groups of proteins manifested significant differences in their expression with respect to risk stratification (Figure 3). In particular, ZA2G, FCN3, CFAB, CLUS, CERU, APOE, APOA4, APOA1, ANT3, AMBP, A1AT, VTB, ACTG, ACTB and SAA molecules appeared to discriminate between low and high-risk leukemias, irrespectively of the tissue of origin; BM or PB. Simultaneously, parallel analysis of karyotype profile concerning protein expression revealed that APOA1, TTHY, VTDB, CERU, CLUS, CFAB, FCN3, HEMO, KNG1, THRB and TRFE proteins could discriminate between a normal and aberrant karyotype (Figure 4). It is apparent that FCN3, CFAB, CLUS, CERU, and APOA1 molecules are commonly significant relating to risk and karyotype.

**Table 2:** Protein expression levels and GO annotation

**A**						
	**BMC/ALL**	**PBC/ALL**	**PBP/HR-ALL**	**PBP/LR-ALL**	**BMP/HR-ALL**	**BMP/LR-ALL**
A1AT	0.93	0.00	0.00	0.00	1.34	0.97
A2MG	0.96	0.00	0.00	0.00	0.00	0.57
ACTB	1.62	0.00	0.00	0.00	0.00	0.00
ACTB	0.00	0.86	0.00	0.00	-0.53	0.00
ACTC	0.00	0.00	0.00	0.00	0.97	0.00
ACTG	0.00	0.00	0.00	0.00	-0.63	0.00
ACTS	0.00	0.00	0.00	0.00	0.87	0.00
AFAM	0.00	0.00	0.00	0.00	0.00	-1.00
AFM	0.00	0.00	0.00	-1.49	0.00	0.00
AMBP	0.00	0.00	0.57	0.00	0.86	0.00
ANGT	0.00	0.00	0.00	0.00	0.00	1.58
ANT3	0.00	0.00	0.00	0.00	1.00	0.75
APOA1	1.03	1.67	0.00	0.00	1.20	0.00
APOA4	0.00	0.00	0.00	0.00	0.91	0.00
APOC2	0.00	0.00	0.00	0.00	0.82	1.50
APOE	0.00	0.00	0.00	0.00	1.36	1.05
BICR1	0.00	0.00	0.00	0.00	0.00	0.88
CALL5	0.00	0.00	0.00	0.00	1.34	0.00
CATA	0.00	1.49	0.00	0.00	0.00	0.00
CERU	1.37	1.58	0.00	0.00	0.70	0.00
CFAB	0.00	0.00	0.00	0.00	1.21	1.27
CH60	0.00	0.00	0.00	0.00	0.00	0.00
CLUS	0.00	0.00	0.00	0.00	0.45	0.00
ENOA	0.00	0.00	0.00	0.00	1.19	1.07
ENOB	0.00	0.00	0.00	0.00	0.00	0.93
FCN3	0.00	0.00	0.00	0.00	1.36	0.00
FHR1	0.00	0.00	0.00	0.00	0.68	0.00
FIBA	0.00	0.00	0.00	0.00	0.89	0.80
FIBB	1.67	1.13	0.00	0.00	1.00	0.70
FIBG	1.42	1.04	0.00	0.00	1.11	1.29
G3P	0.00	0.00	0.00	0.00	0.00	0.61
GELS	0.00	0.00	-1.22	0.00	-0.82	0.00
HEMO	0.93	0.83	0.00	0.00	0.00	1.11
HPT	1.34	1.58	0.00	0.00	0.00	0.90
IGHG1	1.11	1.06	0.00	0.00	0.00	-0.87
IGHG2	1.67	1.00	0.00	0.00	0.00	0.00
KNG1	0.00	0.00	0.00	1.53	0.00	1.30
KPYM	0.00	0.00	0.00	0.00	1.40	1.05
PLMN	0.00	0.00	0.00	0.00	0.00	0.89
PRDX1	0.00	0.00	0.00	0.00	0.00	1.32
PSME1	0.00	0.00	0.00	0.00	0.00	1.00
S10A9	-0.67	-1.27	0.00	0.00	-1.37	-1.65
SAA	1.39	0.89	0.00	0.00	1.40	0.81
THRB	0.00	0.00	0.00	0.00	0.43	0.00
TRFE	1.07	1.41	0.00	0.00	1.53	0.00
TTHY	0.96	0.00	0.00	0.00	1.53	1.06
UBQL1	0.00	0.00	0.00	0.00	-1.11	0.00
VTDB	0.00	0.00	0.00	0.00	0.58	1.31
VTNC	0.00	0.00	0.00	0.00	0.00	-1.04
ZA2G	0.00	0.00	0.00	0.00	1.00	0.97
**B**						
**biological process----triglyceride-rich lipoprotein particle remodeling----GO:0034370**						
C=11;O=4;E=0.01;R=373.25;rawP=1.85e-10;adjP=2.12e-08						
APOE						apolipoprotein E
APOC2						apolipoprotein C-II
APOA4						apolipoprotein A-IV
APOA1						apolipoprotein A-I
**biological process----phospholipid efflux----GO:0033700**						
C=10;O=4;E=0.01;R=410.57;rawP=1.18e-10;adjP=2.12e-08						
APOE						apolipoprotein E
APOC2						apolipoprotein C-II
APOA4						apolipoprotein A-IV
APOA1						apolipoprotein A-I
**biological process----very-low-density lipoprotein particle remodeling----GO:0034372**						
C=11;O=4;E=0.01;R=373.25;rawP=1.85e-10;adjP=2.12e-08						
APOE						apolipoprotein E
APOC2						apolipoprotein C-II
APOA4						apolipoprotein A-IV
APOA1						apolipoprotein A-I
**biological process----reverse cholesterol transport----GO:0043691**						
C=16;O=4;E=0.02;R=256.61;rawP=1.02e-09;adjP=8.77e-08						
APOE						apolipoprotein E
APOC2						apolipoprotein C-II
APOA4						apolipoprotein A-IV
APOA1						apolipoprotein A-I
**biological process----regulation of cholesterol transport----GO:0032374**						
C=20;O=4;E=0.02;R=205.29;rawP=2.71e-09;adjP=1.28e-07						
APOE						apolipoprotein E
APOC2						apolipoprotein C-II
APOA4						apolipoprotein A-IV
APOA1						apolipoprotein A-I
**biological process----regulation of sterol transport----GO:0032371**						
C=20;O=4;E=0.02;R=205.29;rawP=2.71e-09;adjP=1.28e-07						
APOE						apolipoprotein E
APOC2						apolipoprotein C-II
APOA4						apolipoprotein A-IV
APOA1						apolipoprotein A-I
**biological process----plasma lipoprotein particle remodeling----GO:0034369**						
C=21;O=4;E=0.02;R=195.51;rawP=3.34e-09;adjP=1.28e-07						
APOE						apolipoprotein E
APOC2						apolipoprotein C-II
APOA4						apolipoprotein A-IV
APOA1						apolipoprotein A-I
**biological process----macromolecular complex remodeling----GO:0034367**						
C=21;O=4;E=0.02;R=195.51;rawP=3.34e-09;adjP=1.28e-07						
APOE						apolipoprotein E
APOC2						apolipoprotein C-II
APOA4						apolipoprotein A-IV
APOA1						apolipoprotein A-I
**biological process----protein-lipid complex remodeling----GO:0034368**						
C=21;O=4;E=0.02;R=195.51;rawP=3.34e-09;adjP=1.28e-07						
APOE						apolipoprotein E
APOC2						apolipoprotein C-II
APOA4						apolipoprotein A-IV
APOA1						apolipoprotein A-I
**biological process----cholesterol efflux----GO:0033344**						
C=25;O=4;E=0.02;R=164.23;rawP=7.05e-09;adjP=2.43e-07						
APOE						apolipoprotein E
APOC2						apolipoprotein C-II
APOA4						apolipoprotein A-IV
APOA1						apolipoprotein A-I

(A) Summary of protein expression values with respect to samples, stratified as high- or low-risk. The values under each sampling category represent the log2 transformed ratio of the samples evaluated over control samples. The value *0* signifies that the protein was not detected in the respective group of samples (*BMP/HR*, Bone marrow plasma/high risk, *BMC*, Bone marrow cells; *PBC*, Peripheral blood cells; *PBP/HR*, Peripheral blood plasma/high risk; *PBP/LR*, Peripheral blood plasma/low risk; *BMP/LR*, Bone marrow plasma/low risk). (B) Part two includes the respective proteins by using their gene symbol sorted by their function as revealed by gene ontology (GO) analysis.

### Protein classification regarding tissue of origin

**Figure 5:**
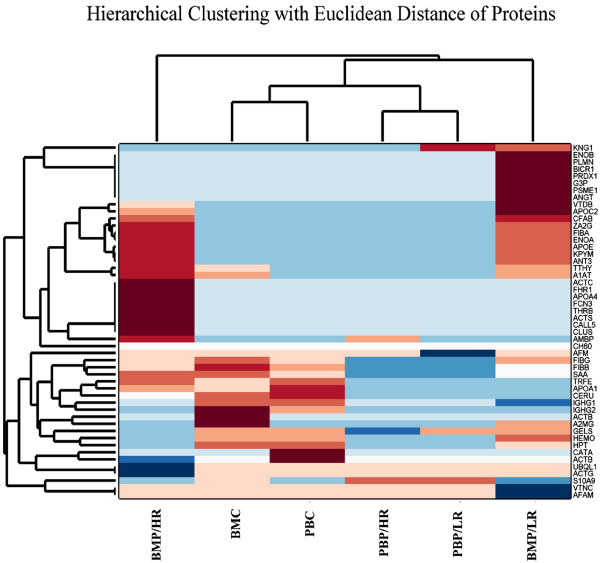
Hierarchical clustering with Euclidean Distance of proteins classified with respect to patient risk (BMP/HR: Bone Marrow Plasma/High Risk, BMC: Bone Marrow Cells, PBC: Peripheral Blood Cells, PBP/HR: Peripheral Blood Plasma/High Risk, PBP/LR: Peripheral Blood Plasma/Low Risk, BMP/LR: Bone Marrow Plasma/Low Risk).

For further information on protein expression profile, hierarchical clustering analysis was used to identify patterns with respect to tissue of origin (Figure 5). Samples were classified in four categories; BMP/HR and BMP/LR were classified in totally opposed groups, following the initial sampling taxonomy. On the other hand, BMC and PBC were grouped collectively as well as PBP/HR with PBP/LR. This indicates that BMC and PBC have very similar profiles on top of PBP/HR and PBP/LR. In particular the latter revealed no key differences in the peripheral blood setting, indicating that the pivotal microenvironment is that of BM.

### PCA of protein expression regarding tissue of origin

**Figure 6:**
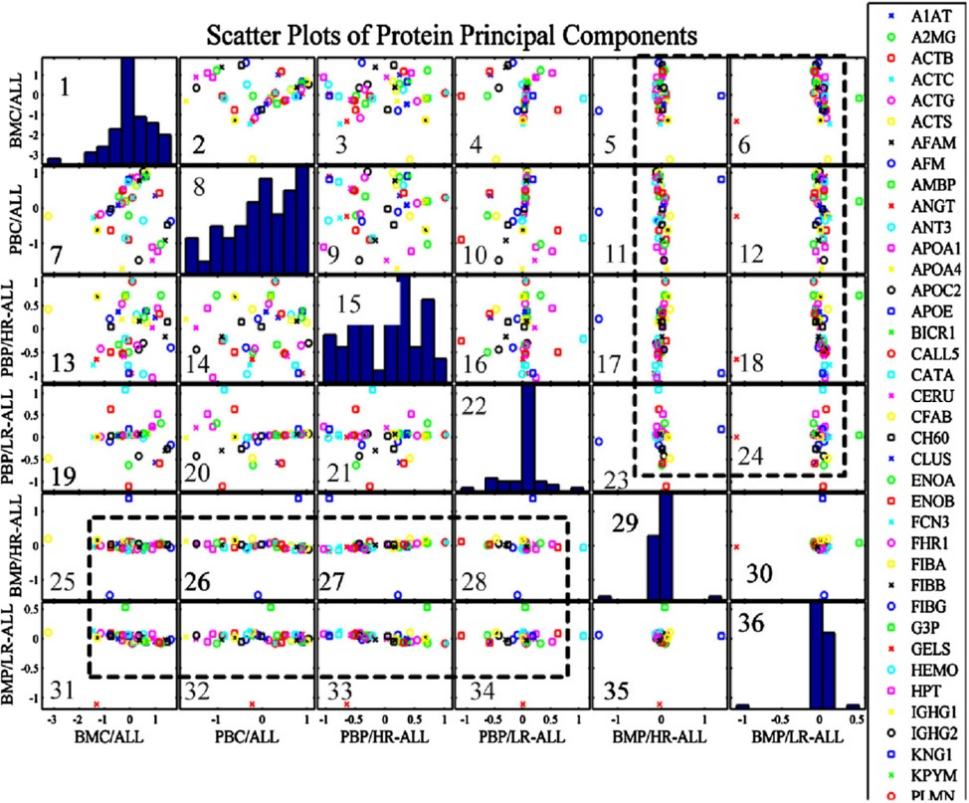
**Scatter plots of Principal Components of protein expression levels.** Linear correlations were revealed between the principal components of BMP/LR and BMP/HR patients and BMC, PBC, PBP/HR and PBP/LR. In addition, between BMP/HR and BMP/LR six proteins appeared to distinguish between the stratified patients: AMBP, AFM, GELS, KNG1, CATA and S10A9 (**BMP/HR**: Bone Marrow Plasma/High Risk, **BMC**: Bone Marrow Cells, **PBC**: Peripheral Blood Cells, **PBP/HR**: Peripheral Blood Plasma/High Risk, **PBP/LR**: Peripheral Blood Plasma/Low Risk, **BMP/LR**: Bone Marrow Plasma/Low Risk, **ALL**: Acute Lymphoblastic Leukemia).

**Figure 7:**
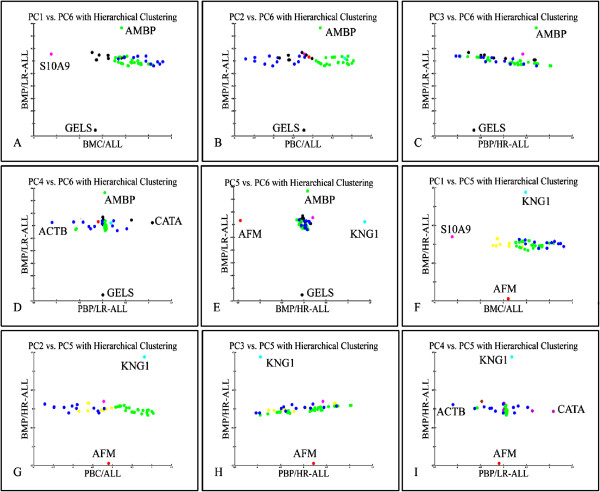
**Principal Components Analysis with Hierarchical Clustering.** Figures **A-I** are a magnification of Figure 6 boxes 25–35. Seven proteins appear to separate low risk from high risk patients and in a tissue specific manner (**BMP/HR**: Bone Marrow Plasma/High Risk, **BMC**: Bone Marrow Cells, **PBC**: Peripheral Blood Cells, **PBP/HR**: Peripheral Blood Plasma/High Risk, **PBP/LR**: Peripheral Blood Plasma/Low Risk, **BMP/LR**: Bone Marrow Plasma/Low Risk, ALL: Acute Lymphoblastic Leukemia).

Examining the scatters of proteins’ principal components, it was obvious that transformed data manifested a linear behavior between BMP/HR and BMP/LR samples compared to the rest sample groups (Figure 6). This is presented in boxes 25–35 with the exception of boxes 30 and 35, where the difference between BM/LR and BM/HR consists of four proteins. In the rest of the boxes, several proteins differentiate beyond the generalized linear expression pattern, indicating a specific role in leukemia. A magnification of those scatter plots is presented in detail to examine their expression patterns (Figure 7). In total, seven proteins discriminated between LR and HR as well as between the tissue of origin (BM or PB) or cellular components (plasma or cell lysate). These proteins included GELS, S10A9, AMBP, ACTB, CATA, AFM and KNG1 (Additional file 3: Figure S5). The diagrams indicated that the principal components of most proteins could be grouped in a uniform formation, whilst several other proteins emerged as outliers. Subsequently, these proteins might be employed to discriminate between the leukemic cell tissue of origin at diagnosis (BM or PB) or the cellular components (cell lysate or plasma).

### Survival analysis

**Figure 8:**
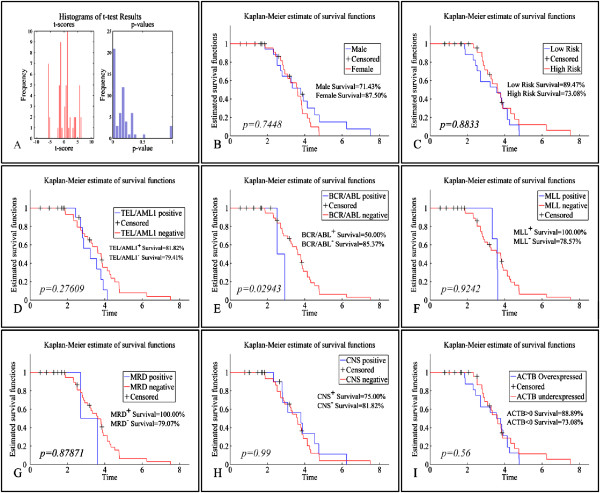
**Kaplan-Meier survival analysis. A:** Overall survival curves of patients according to APOA1, CERU, FIBB, FIBG, IGHG1, IGHG2, S10A9, SAA, TTHY, A2MG, APOA1, ACTB, CATA, CERU, FIBB, FIBG, HPT, HEMO, IGHG1, S10A9 (*p<0.01*); Survival rates between: **B:** males and females; **C:** low- and high-risk cases; **D:***TEL/AML1* positive and negative patients; **E:***BCR/ABL* positive and negative patients; **F:***MLL* positive and negative patients; **G:** MRD positive and negative patients; **H:** CNS positive and negative patients; **I:** Indicatevely, a survival curve for the ACTB protein, which did not manifest significant differences in survival rates. The same was true for all proteins under study.

The OS was estimated at 88.8%. A two-tailed t-test was used to determine the significance in protein expression levels between alive and deceased patients (Figure 8A). OS was found significant regarding APOA1, CERU, FIBB, FIBG, IGHG1, IGHG2, S10A9, SAA, TTHY, A2MG, APOA1, ACTB, CATA, CERU, FIBB, FIBG, HPT, HEMO, IGHG1, and S10A9 molecules (*p < 0.01*). Therefore, these proteins might play a significant role in leukemia progression and outcome. Additionally, *BCR/ABL* proved to manifest significant difference with respect to survival (Figure 8E). The rest of the clinical factors did not present significant differences with respect to OS. However, individual proteins did not manifest significant results concerning the survival of leukemic patients, which supports the hypothesis that it is not the effect of an isolated protein, but rather the coordinated regulatory network of proteins. Although OS did not appear to be dependent on individual protein levels, the fact that several proteins are differentially expressed between alive and deceased patients points towards this very fact: it is the result of a network and combination of functions as to leukemia outcome. This raises the possibility that oncogenesis is multifactorial. OS rates have been calculated as the percentage of alive or deceased patients from the day of diagnosis to the present day. In addition, LFS showed more significant confidence levels than OS, yet none of the clinicopathological factors appeared to influence significantly (p < 0.05) the LFS rates (data not shown).

### Network view

Blue connections are inferred by phylogenetic co-occurrence, whereas light blue lines indicate database evidence. The line thickness is a rough indicator of the strength of the association. The visualizations show the predicted association between the proteins detected in the samples of leukemic and non-leukemic patients (Additional file 4: Figure S2).

### Gene ontology (GO) analysis of expressed proteins

Functional analysis of expressed proteins revealed that APOE, APOC2, APOA4 and APOA1 proteins participate in cholesterol regulation and lipoprotein modeling (Additional file 5: Figure S3). Of note, APOA1 appeared previously to discriminate between LR and HR leukemias as well as between normal and aberrant karyotype.

### Pathway analysis of expressed proteins

Further analysis included pathway participation for the expressed proteins. Interestingly, it appeared that the same proteins as in GO analysis, appeared to participate in the statin pathway, which includes cholesterol regulation and lipoprotein modeling (Additional file 6: Figure S4). More specifically, APOE, APOC2, APOA4 and APOA1 participated in the statin pathway.

### Western blot analyses

**Figure 9:**
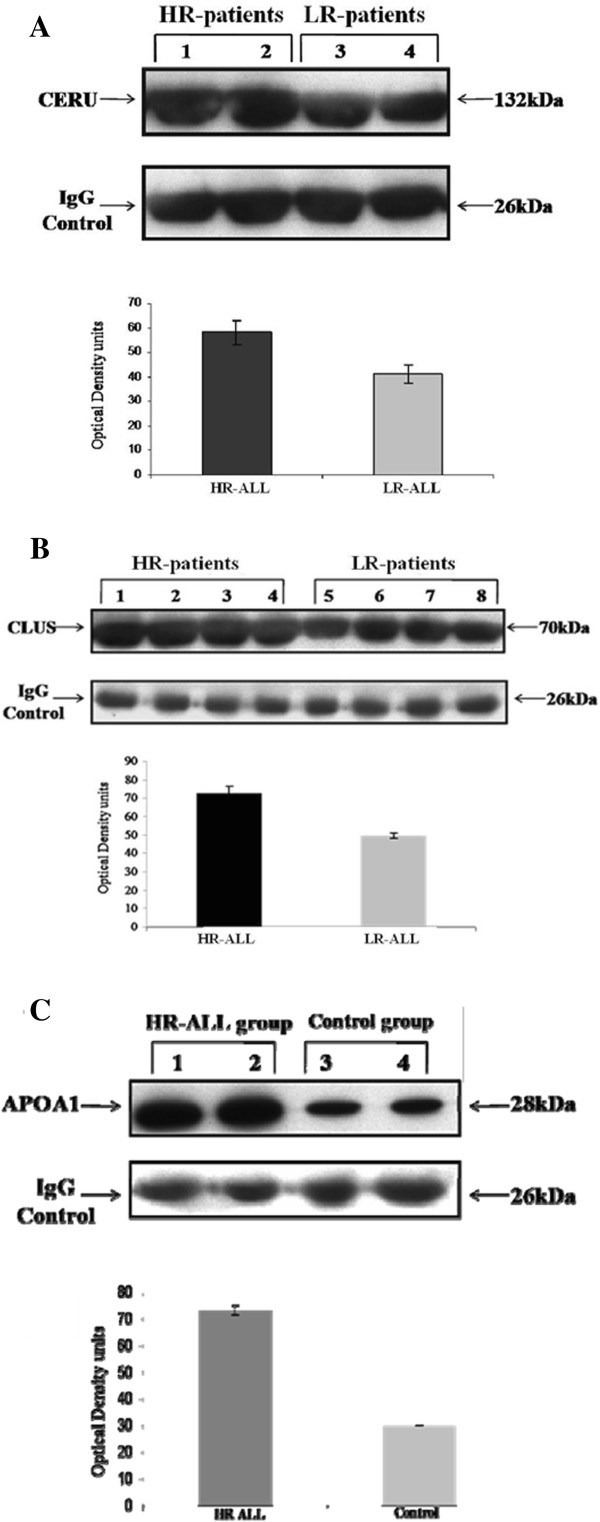
**Western blot analysis of ceruplasmin expression in HR-ALL patients (Lanes 1 and 2) and LR-ALL patients (Lanes 3 and 4).** Also, quantification of ceruloplasmin content using scanning densitometry. Each bar represents the mean Optical Density ± SD of three independent experiments. Differences were significant at the level of p < 0.01 **(A)**. Western blot analysis of clusterin expression in HR-ALL patients (Lanes 1–4) and LR-ALL patients (Lanes 5–8). Also, quantification of ceruloplasmin content using scanning densitometry. Each bar represents the mean Optical Density ± SD of three independent experiments. Differences were significant at the level of p < 0.01 **(B)**. Western blot analysis of APOA1 expression in HR-ALL patients (Lanes 1 and 2) and non-leukemic patients (Lanes 3 and 4). Also, quantification of APOA1 content using scanning densitometry. Each bar represents the mean Optical Density ± SD of three independent experiments. Differences were significant at the level of p < 0.01 **(C)**.

To verify previous findings, Western blot analysis was ascertained. The elevated expression of CERU and CLUS in HR-patients as compared to LR-patients, was confirmed (Figure 9A and Figure 9B, respectively). In addition, the upregulation of APOA1 as compared to control samples was verified (Figure 9C).

## Discussion

Since therapeutic interventions are suboptimal, ongoing research has attempted to offer complementary insights into the understanding of pediatric ALL by various approaches, including clinical and biological variables [32]. In the current study, investigation was centered on the elucidation of differential protein patterns between non-leukemic, LR- and HR-ALL pediatric patients, in order to examine whether certain proteins or groups of proteins might afford useful indicators of leukemia aggressiveness or patients’ outcome.

The proteomic analysis of plasma revealed significant consistencies between BM and PB samples. There was an overlap of thirteen proteins between the BM and PB plasma samples from both LR- and HR-ALL patients with diverse clinical outcomes. The majority of these proteins were found up-regulated as compared to control samples. Regarding HR-ALL patients (both in BMP and PBP), there was a significant decrease in levels of expression of GELS when compared with samples derived from non-leukemic patients, suggesting the potential prognostic value of this molecule as a suppressor protein in aggressive ALL cases. GELS, a Ca^++^ regulated actin filament severing, capping and nucleating protein affects major cytoskeletal changes during differentiation and carcinogenesis and has also been considered as a strong indicator of apoptosis [33,34]. Several reports have suggested the prognostic role of GELS in various cancer types, including breast cancer [33], brain tumor astrocytoma [35] and childhood AML [21]. Regarding AMBP expression, up-regulation was observed in the HR-ALL cases (both in BMP and PBP) when compared to the control group, proposing a potential contributing role in pediatric ALL severity. Our observations are in line with previous work also recommending AMBP’s involvement in carcinogenesis [36].

Remaining in the HR-risk-group of patients, elevated levels of CERU, CLUS and FCN3 were monitored when compared to the LR-ALL. We have previously provided evidence that CERU and CLUS displayed altered expression in BM plasma samples obtained from pediatric patients with myelodysplastic syndrome (MDS), therapy related-AML (t-AML) [37] and *de novo* AML [21]. CLUS plays important role in the majority of biological phenomena including cell proliferation and apoptosis as well as in a variety of diseases including cancer [38]. More specifically it has been linked to prostate [39], pancreatic [40], colon [41], ovarian [42], colorectal cancers [43] and may play an important role in breast cancer initiation and development [44,45]. Moreover, recent studies suggested the potential role of FCN3 in ovarian and prostate cancers [46,47]. Taken together, in our study, the identification of all three proteins in HR-ALL patients indicates a potential contributing value in adjectival leukemia and a prospect to afford candidate biomarkers for leukemia progression. However, it is noteworthy that both CERU and CLUS are acute phase proteins, which fluctuate in response to inflammation and subsequently their enhanced expression might not be ALL specific, since at ALL diagnosis, the vast majority of patients harbor microbial infections.

Regarding the LR-ALL patient group, a significant suppression in the expression of AFAM was detected in all BM plasma samples from patients with dissimilar disease outcomes when compared to the control group. Interestingly, previous reports also showed decreased expression of AFAM in patients with ovarian cancer indicating its potential use as an additional disease biomarker [46,48,49]. Therefore, overall, these observations support the notion that AFAM could be considered as a candidate protein marker not only for ovarian cancer, but for childhood leukemia as well. However, no additional reports were found relating this molecule to pediatric ALL. Remaining in the same risk group of patients, KNG1 was found consistently overexpressed in both BM and PB plasma specimens derived from LR-ALL patients as compared to control samples, while it was not detected in the HR-ALL group (signifying its value as 0), suggesting a possible role in leukemogenesis. On the contrary, in previous reports, lowered expression of KNG1 was detected in various types of cancers including gastrointestinal, breast and cervical cancers, which has been attributed to its contribution to the survival of cancer cells. KNG1 is considered to have anti-angiogenic properties and inhibitory action on the proliferation of endothelial cells [50]. Yet, it was interesting to see that survival rates did not appear to correlate significantly with the differentially expressed proteins. This could be supported by the hypothesis that survival, as well as the disease itself, is not the result of one particular protein but rather of the interaction between several different proteins. Overexpression of PLMN and down-regulation of VTNC were observed in both BM plasma specimens from LR-ALL group of patients when compared with corresponding samples obtained from the control groups. PLMN is considered a glycoprotein, associated with the development of thrombosis, which has been found overexpressed in several life-threatening diseases and has been correlated with dismal outcomes [51]. In general, for pediatric malignancies there is an enhanced risk of thrombosis frequently as a result of malfunctions of the endothelium of blood vessels in the ALL patients. In addition, previous reports [52] documented that in malignant cells, elevated levels of PLMN activators have been observed. These proteases convert inactive PLMN into active plasmin and degrade a variety of proteins including VTNC during invasion and metastasis. This VTNC degradation, also finds application in our study, since decreased levels of VTNC were identified only in BM and PB plasma samples from ALL patients, when compared to non-leukemic patients. Therefore, it is likely that these molecules might represent additional contributors to leukemogenesis. Importantly, protein interaction network analysis revealed a strong association between PLMN and VTNC.

Elevated levels of VTDB were observed in both BM plasma samples from LR- and HR-ALL patients when compared to non-leukemic patients. The current data adds to the growing body of evidence indicating a potential association between VTDB and oncogenesis. We have previously documented a contributing role of VTDB in pediatric t-AML [37] and secondary AML following MDS [21]. In addition, there is recent considerable data supporting the potential role of VTDB status in cancer [53-56], however its role in cancer remains controversial [57].

Concerning cell lysates, the protein patterns obtained during screening did not reveal any significant disparities between BM and PB specimens or LR- and HR-ALL cases or patients’ clinical outcomes. The most imperative protein with significantly higher levels of expression in PB cell lysates samples from LR- and HR-ALL cases was the CATA antioxidant enzyme. Antioxidant enzymes constitute the major cellular protection against oxidants and therefore, they have been previously associated with carcinogenesis and tumor progression [58]. According to el Bouhtoury et al. [59], CATA frequently displays inadequate levels of expression in several malignancies. However, our findings are in line with several previous studies suggesting up-regulation of catalase in malignant mesothelioma tumors [60], childhood and adult *de novo* AML [21,58], as well as childhood t-AML [37].

The relatively elevated expression of high abundance proteins in plasma composes a major challenge, since they might mask low abundance proteins of interest [61]. For this reason, removal of these proteins, constituting approximately 80% of the protein content was performed, in order to unmask lower-abundance proteins that might play essential roles in the prognostication or therapeutic determinations of the disease. Of note, complete depletion of high abundance proteins was not feasible however, the vast majority of them were successfully removed. Following removal, a variety of molecules were identified. Most proteins detected have been previously associated with cancer [62-64]. The most imperative protein that was found up-regulated in all BM samples derived from LR-ALL patients, when compared to the controls, was the BICR1 molecule. Bicaudal D1 is known to be involved in mRNA and Golgi-endoplasmic reticulum vacuolar transport [65]. However, recent studies suggested its potential use as a potent suppressor of the protease-activated-receptor-1 (PAR-driven), which plays a central role in cancer [66]. More importantly, BIRC1 has also been associated with telomere length variation in humans [67]. Subsequently, the identification of this molecule in pediatric ALL cases might suggest an early telomere dysfunction in these children and therefore could afford a potential biomarker for cancer therapeutics.

We also endeavored to investigate whether there was a positive correlation between specific protein signatures in LR- or HR-ALL patients, presented with recurrent cytogenetic abnormalities and corresponding groups of patients bearing no cytogenetic aberrations. However, no direct linkage was detected. Our findings are comparable with data obtained from a similar study we have previously performed on childhood acute myelogenous leukemia (AML) [21]. Again this could probably suggest that cytogenetic abnormalities do not afford a sole leukemia leukemia, although there is a large debate on the aspects of leukemogenesis. Subsequently, leukemia might result from a complex network of events that can be understood only through representation of such networks [68].

Following bioinformatics analysis it appeared that some proteins could be key regulators in the distinction between low- and high-risk leukemias. Collectively, these proteins included CLUS, CERU, APOE, APOA4, APOA1, GELS, S10A9, AMBP, ACTB, CATA and AFAM. It seems that they played multiple roles in leukemia as well as to the mechanism that controls the relationship between BM and PB. PCA analysis revealed a perfect linear relationship between BMP/HR and BMP/LR samples and all remaining sampling groups, indicating that the protein profile could be similar in leukemic sub-populations; regarding the body location. However, the expression profiles of a certain group of proteins were altered significantly, signifying their role as key regulators in ALL. In addition, several of these proteins are extracellular factors localized in PB, which indicates them potential diagnostic or prognostic factors due to the easiness they can be detected. KNG1 especially is a protease inhibitor, which hints us towards two aspects: protein regulation and metabolism. Metabolism is a candidate factor for leukemia resistance to therapy. It has been previously proposed the relationship of proteasome inhibition and sensitivity to glucocorticoid therapy, which involves the recycling of proteins for metabolic reasons [69]. Therefore, the up-regulation of a protease inhibitor in LR leukemic patients could point towards similar direction. Additionally, it is noteworthy that proteins manifesting a role in the studied phenomenon are involved with lipoprotein modeling. It is known that lipid metabolism is altered during tumor progression with cholesterol being accumulated in tumor cells [70]. It remains controversial whether this is due to metabolic effects or just a malfunction of tumor cells. APOA1, the major protein component of high-density lipoprotein (HDL) was up-regulated in both LR- and HR-leukemias, verifying a lipid metabolic derangement of HDL [71].

Herein, our findings support the growing role of certain proteins in pediatric ALL. More specifically, it became evident that the differential expression of VTNC and PLMN possibly contributed to leukemogenesis. In addition, KNG1 and FCN3 potentially served as distinctive biomarkers for leukemia aggressiveness, whereas GELS played a restraining role as a suppressor protein in HR-ALL cases. CLUS, CERU, APOE, APOA4, APOA1, GELS, S10A9, AMBP, ACTB, CATA and AFAM might serve as potential diagnostic or prognostic markers distinctive between LR- and HR-leukemic patients, however, this requires additional investigations. Moreover, BICR1 could probably afford a significant biomarker for pediatric ALL therapeutics. Consequently, taken together, the proteins identified, although not sturdily predictive of patients’ outcome, still might compose promising targets related to pediatric ALL progression for therapeutic intervention.

## Abbreviations

AFAM: Afamin; ALL: Acute lymphoblastic leukemia; AMBP: Alpha-1-microglobulin/bikunin precursor; AML: Acute myeloid leukemia; APOA1: Apolipoprotein A1; BICR1: Bicaudal D-related protein 1; BM: Bone marrow; CATA: Catalase; CD5L: CD5 antigen; CERU: Ceruloplasmin; CLUS: Clusterin; FCN3: Ficolin-3; FINC: Fibronectin; GELS: Gelsolin; HR: High risk; iFISH: Interphase fluorescence in situ hybridization; KNG1: Kininogen 1; LR: Low risk; MDS: Myelodysplastic syndrome; PB: Peripheral blood; PLMN: Plasminogen; S10A9: Protein S100-A9; SAA: Serum amyloid A; t-AML: Therapy-related AML; VTDB: Vitamin D-binding protein; VTNC: Vitronectin.

## Competing interests

The authors declare no conflict of interest.

## Authors’ contribution

MB organized the research plan, performed the experiments and drafted the manuscript; GIL performed data analysis and participated in manuscript writing; KV performed MS experiments; KK provided samples and clinical data; GTT contributed to the experimental design and provided research facilities; FT-S coordinated the study, participated to its design and contributed to writing. All authors read and approved the manuscript.

## Supplementary Material

Additional file 1: Figure S1 Summary of patient clinical data: mean age of males and females (A), mean white blood cell count in males and females (B), number of children diagnosed (C), mean white blood cell count with respect to diagnosis (D), number of children with known chromosomal abberations (E), mean white blood cell count with respect to chromosomal abberations (F). Click here for file

Additional file 2: Table S1 Differentially expressed proteins in BM plasma derived from non-leukemic , LR- and HR- ALL patients. Table S2A: Differentially expressed proteins in PB plasma derived from non leukemic and ALL patients (common between LR- and HR). Table S2B: Differentially expressed proteins in PB plasma derived from HR-ALL and LR-ALL patients. Table S3: Differentially expressed proteins in BM and PB cell lysates derived from ALL patients. Table S4: Differentially expressed pre-fractionated proteins in BM and PB plasma derived from ALL patients. Click here for file

Additional file 3: Figure S5 Indicative diagrams from the Principal Components in Figure 7 with linearity fittings (blue line) and 95% prediction bounds (dashed lines). The red “plus” signs indicate the values that have been excluded and consist of those values, as presented in Figure 7, that separate tissue of sampling of leukemic cells with respect to proteins. Click here for file

Additional file 4: Figure S2 Expression Profiling Diagrams of the BM plasma proteins detected in Figure 1A: SAA1; serum amyloid A protein, CLU; clusterin, AMBP; AMBP protein precursor, AZGP1; zinc-alpha-2-glycoprotein precursor, F2; prothrombin, APOE; apolipoprotein E, ACTB; actin cytoplasmic 1, TTR; transthyretin, APOA1; apolipoprotein A-I precursor, S100A9; protein S100A9, ACTG1; actin cytoplasmic 2, CP; ceruloplasmin, GC; vitamin D-binding protein, GSN; gelsolin, APOA4; apolipoprotein A-IV, ENSG00000166285; complement factor B, SERPINC1; antithrombin-III, SERPINA1; alpha-1-antithrypsin, FCN3; ficolin-3. Figure 1B: SAA1; serum amyloid A protein, HP; haptoglobin, AZGP1; zinc-alpha-2-glycoprotein precursor, APOE; apolipoprotein E, HPX; hemopexin, TTR; transthyretin, AGT; angiotensinogen, S100A9; protein S100A9, AFM; afamin, GC; vitamin D-binding protein, KNG1; kininogen-1, FGG; fibrinogen gamma chain, APOC2; apolipoprotein C-II, SERPINC1; antithrombin-III, A2M; alpha-2 macroglobulin, SERPINA1; alpha-1-antithrypsin. Figure 1C: GC; vitamin D-binding protein, KNG1; kininogen-1, AMBP; AMBP protein precursor, AZGP1; zinc-alpha-2-glycoprotein precursor, GSN; gelsolin, PLG; plasminogen, VTN; vitronectin, IGHM; immunoglobulin heavy chain C, A2M; alpha-2 macroglobulin, CD5L, CD5 antigen. Click here for file

Additional file 5: Figure S3 Gene Ontology of common proteins in different samplings. Cholesterol regulation and lipoprotein remodeling appeared to be the most significant biological function in which the proteins participate. The figure was constructed using WebGestalt web-tool [24]. Click here for file

Additional file 6: Figure S4 Four proteins, significant with respect to risk stratification, participate in the statin pathway, which is involved in cholrsterol regulation and lipoprotein remodeling, as it also appeared from the GO analysis. The figure was constructed using WebGestalt web-tool [24]. Click here for file
