# Supplementary material for: Protein biomarkers distinguish between high- and low-risk pediatric acute lymphoblastic leukemia in a tissue specific manner
Source: J Hematol Oncol. 2013 Jul 12;6:52. doi: 10.1186/1756-8722-6-52 (PMC3717072; doi:10.1186/1756-8722-6-52)
Supplement: Additional file 1: Figure S1 — Summary of patient clinical data: mean age of males and females (A), mean white blood cell count in males and females (B), number of children diagnosed (C), mean white blood cell count with respect to diagnosis (D), number of children with known chromosomal abberations (E), mean white blood cell count with respect to chromosomal abberations (F). [file 1756-8722-6-52-S1.pdf]

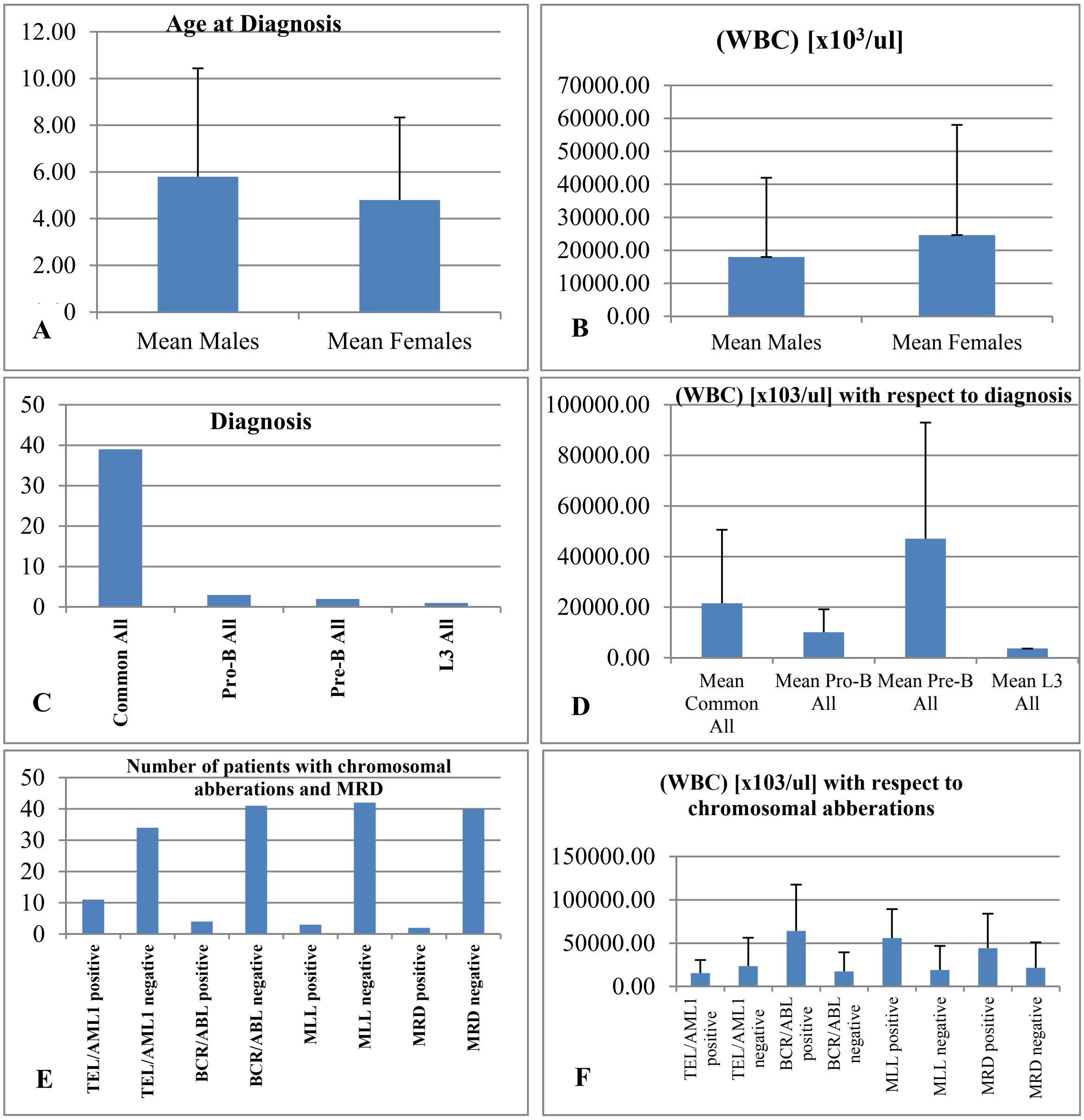

Supplementary Figure 1. Summary of patient clinical data: mean age of males and females (**A**), mean white blood cell count in males and females (**B**), number of children diagnosed (**C**), mean white blood cell count with respect to diagnosis (**D**), number of children with known chromosomal abberations (**E**), mean white blood cell count with respect to chromosomal abberations (**F**).
