# Supplementary material for: Protein biomarkers distinguish between high- and low-risk pediatric acute lymphoblastic leukemia in a tissue specific manner
Source: J Hematol Oncol. 2013 Jul 12;6:52. doi: 10.1186/1756-8722-6-52 (PMC3717072; doi:10.1186/1756-8722-6-52)
Supplement: Additional file 3: Figure S5 — Indicative diagrams from the Principal Components in Figure 7 with linearity fittings (blue line) and 95% prediction bounds (dashed lines). The red “plus” signs indicate the values that have been excluded and consist of those values, as presented in Figure 7, that separate tissue of sampling of leukemic cells with respect to proteins. [file 1756-8722-6-52-S3.pdf]

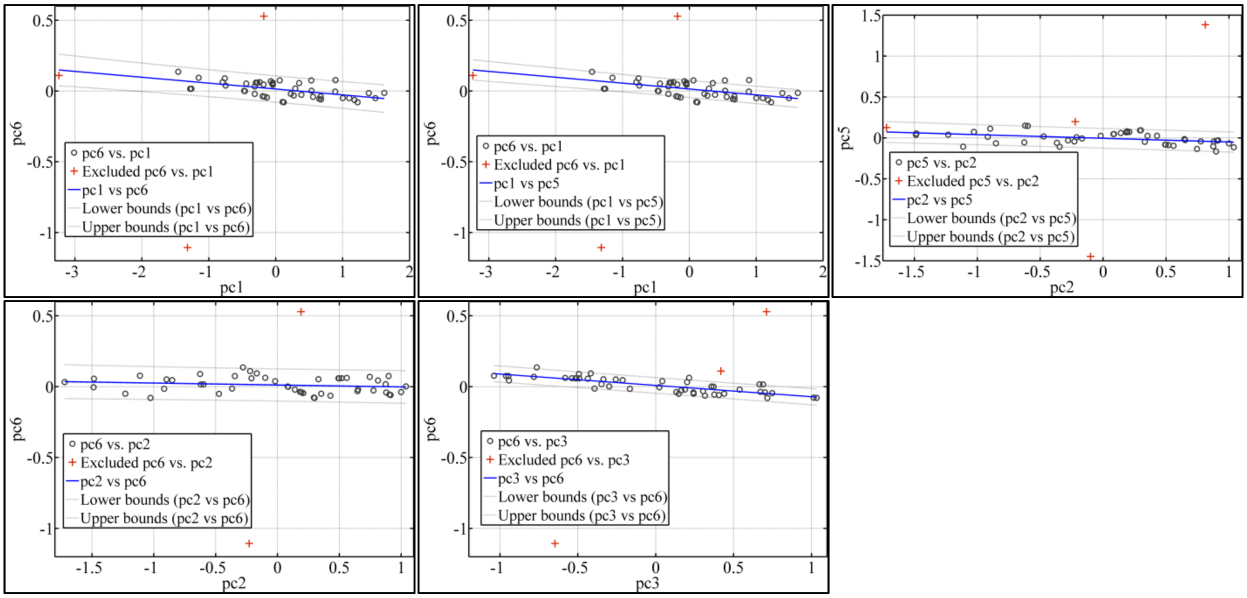


**Supplementary Figure 5**. Indicative diagrams from the Principal Components in Figure 7 with linearity fittings (blue line) and 95% prediction bounds (dashed lines). The red "plus" signs indicate the values that have been excluded and consist of those values, as presented in Figure 7, that separate tissue of sampling of leukemic cells with respect to proteins.
