# Supplementary material for: Protein biomarkers distinguish between high- and low-risk pediatric acute lymphoblastic leukemia in a tissue specific manner
Source: J Hematol Oncol. 2013 Jul 12;6:52. doi: 10.1186/1756-8722-6-52 (PMC3717072; doi:10.1186/1756-8722-6-52)
Supplement: Additional file 4: Figure S2 — Expression Profiling Diagrams of the BM plasma proteins detected in Figure 1A: SAA1; serum amyloid A protein, CLU; clusterin, AMBP; AMBP protein precursor, AZGP1; zinc-alpha-2-glycoprotein precursor, F2; prothrombin, APOE; apolipoprotein E, ACTB; actin cytoplasmic 1, TTR; transthyretin, APOA1; apolipoprotein A-I precursor, S100A9; protein S100A9, ACTG1; actin cytoplasmic 2, CP; ceruloplasmin, GC; vitamin D-binding protein, GSN; gelsolin, APOA4; apolipoprotein A-IV, ENSG00000166285; complement factor B, SERPINC1; antithrombin-III, SERPINA1; alpha-1-antithrypsin, FCN3; ficolin-3. Figure 1B: SAA1; serum amyloid A protein, HP; haptoglobin, AZGP1; zinc-alpha-2-glycoprotein precursor, APOE; apolipoprotein E, HPX; hemopexin, TTR; transthyretin, AGT; angiotensinogen, S100A9; protein S100A9, AFM; afamin, GC; vitamin D-binding protein, KNG1; kininogen-1, FGG; fibrinogen gamma chain, APOC2; apolipoprotein C-II, SERPINC1; antithrombin-III, A2M; alpha-2 macroglobulin, SERPINA1; alpha-1-antithrypsin. Figure 1C: GC; vitamin D-binding protein, KNG1; kininogen-1, AMBP; AMBP protein precursor, AZGP1; zinc-alpha-2-glycoprotein precursor, GSN; gelsolin, PLG; plasminogen, VTN; vitronectin, IGHM; immunoglobulin heavy chain C, A2M; alpha-2 macroglobulin, CD5L, CD5 antigen. [file 1756-8722-6-52-S4.pdf]

A.

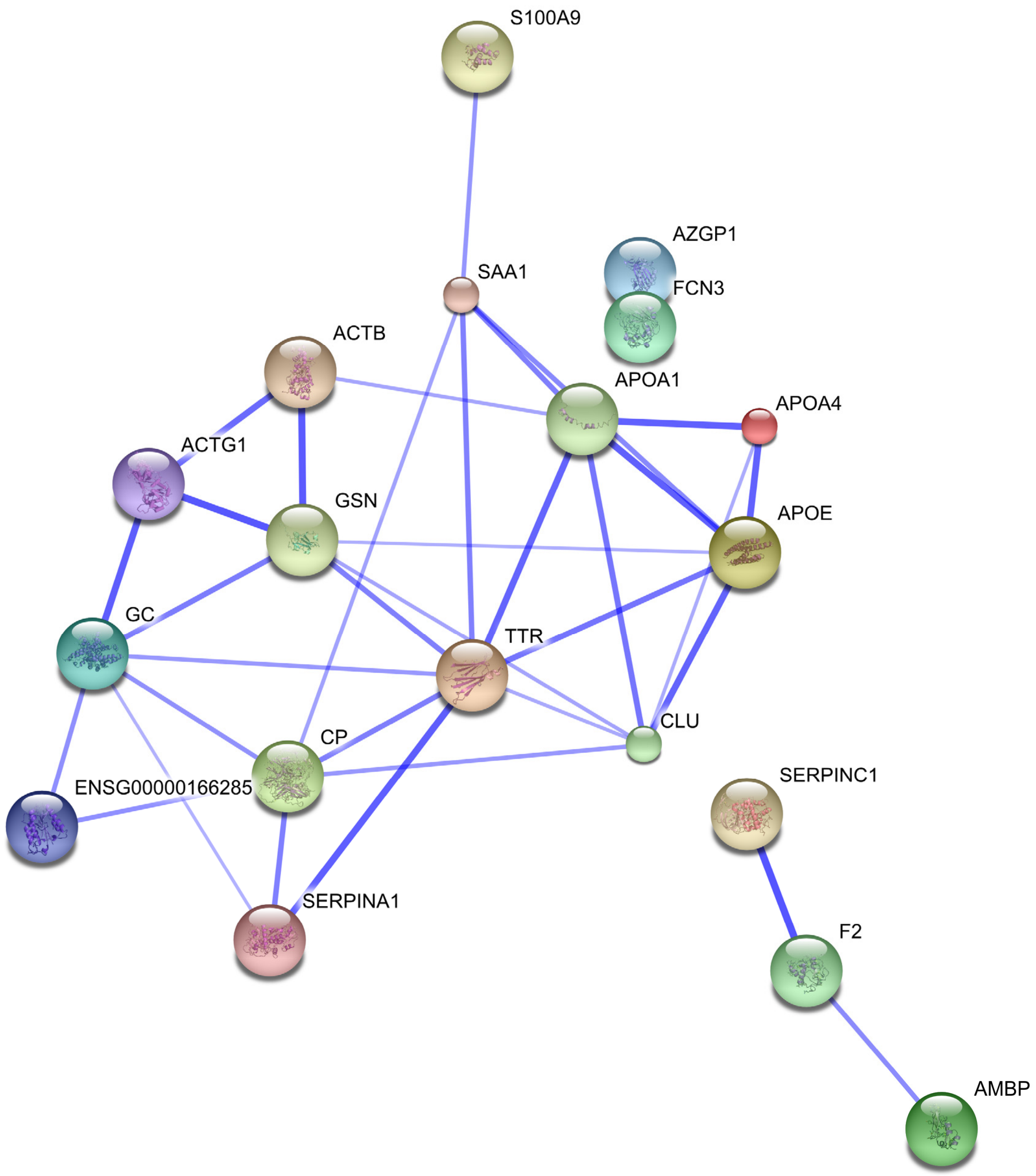

Supplementary Figure 2. Expression Profiling Diagrams of the BM plasma proteins detected in **Figure 1A**: SAA1; serum amyloid A protein, CLU; clusterin, AMBP; AMBP protein precursor, AZGP1; zinc-alpha-2-glycoprotein precursor, F2; prothrombin, APOE; apolipoprotein E, ACTB; actin cytoplasmic 1, TTR; transthyretin, APOA1; apolipoprotein A-I precursor, S100A9; protein S100A9, ACTG1; actin cytoplasmic 2, CP; ceruloplasmin, GC; vitamin D-binding protein, GSN; gelsolin, APOA4; apolipoprotein A-IV, ENSG00000166285; complement factor B, SERPINC1; antithrombin-III, SERPINA1; alpha-1-antithrypsin, FCN3; ficolin-3.

B

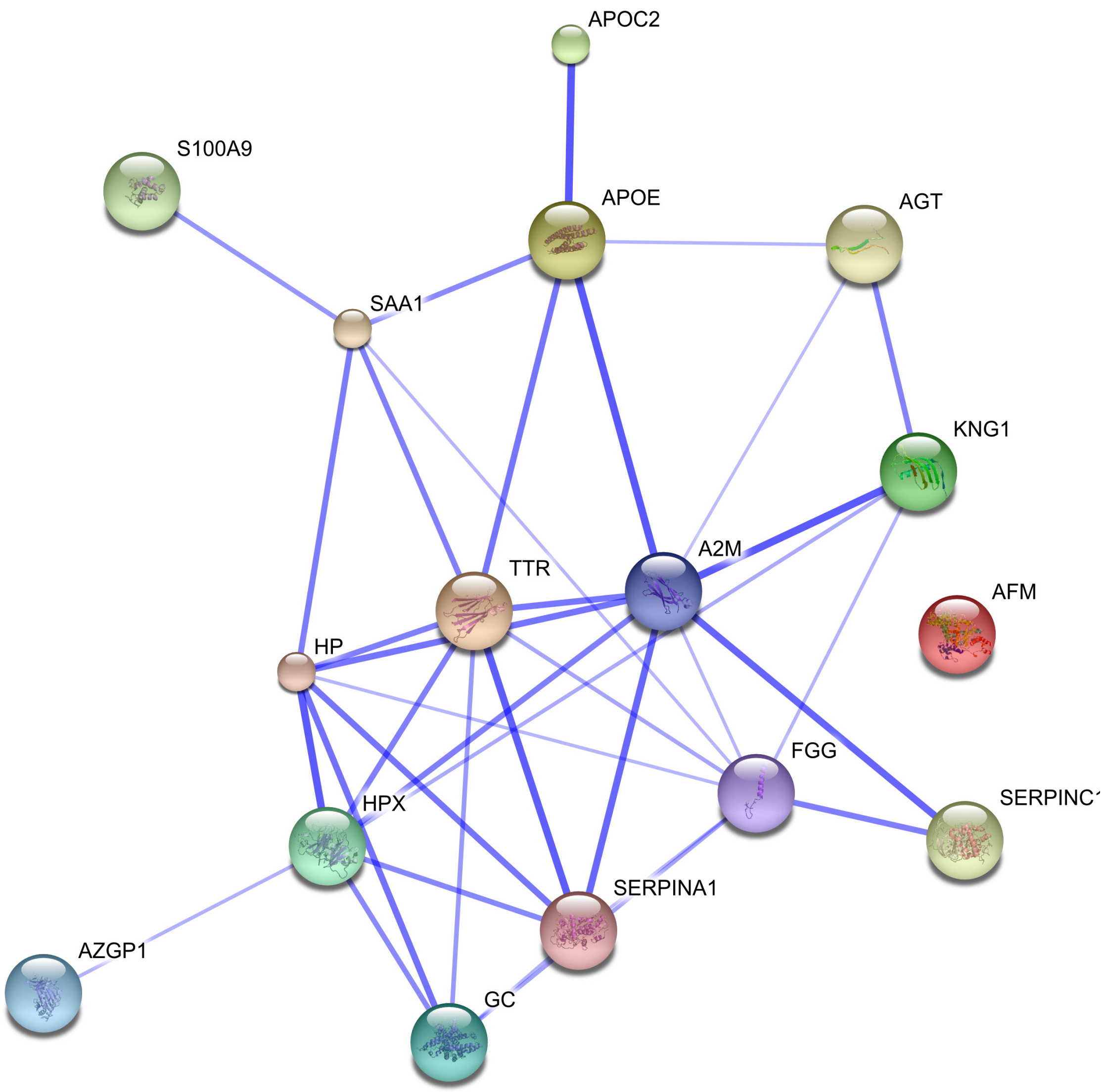

Supplementary Figure 2 (continued). Expression Profiling Diagrams of the BM plasma proteins detected in **Figure 1B**: SAA1; serum amyloid A protein, HP; haptoglobin, AZGP1; zinc-alpha-2-glycoprotein precursor, APOE; apolipoprotein E, HPX; hemopexin, TTR; transthyretin, AGT; angiotensinogen, S100A9; protein S100A9, AFM; afamin, GC; vitamin D-binding protein, KNG1; kininogen-1, FGG; fibrinogen gamma chain, APOC2; apolipoprotein C-II, SERPINC1; antithrombin-III, A2M; alpha-2 macroglobulin, SERPINA1; alpha-1-antithrypsin.

C

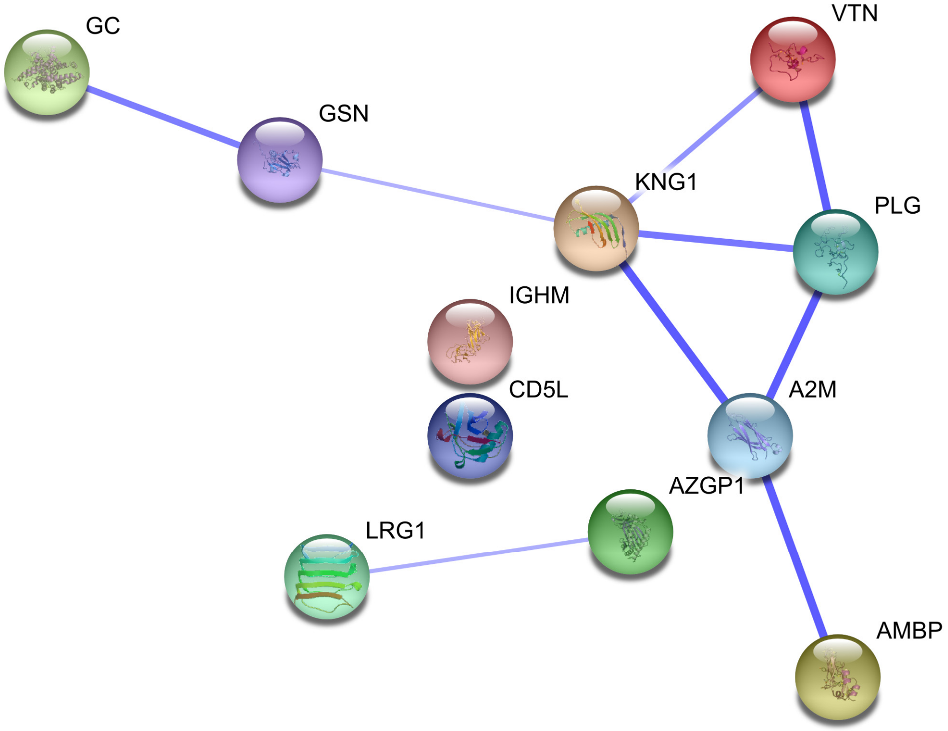

Supplementary Figure 2 (continued). Expression Profiling Diagrams of the BM plasma proteins detected in **Figure 1C:** GC; vitamin D-binding protein, KNG1; kininogen-1, AMBP; AMBP protein precursor, AZGP1; zinc-alpha-2-glycoprotein precursor, GSN; gelsolin, PLG; plasminogen, VTN; vitronectin, IGHM; immunoglobulin heavy chain C, A2M; alpha-2 macroglobulin, CD5L, CD5 antigen.
