# Supplementary figures and images for: Protein biomarkers distinguish between high- and low-risk pediatric acute lymphoblastic leukemia in a tissue specific manner
Source: J Hematol Oncol. 2013 Jul 12;6:52. doi: 10.1186/1756-8722-6-52 (PMC3717072; doi:10.1186/1756-8722-6-52)

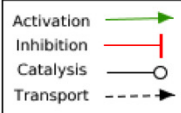

24].

Supplement: Additional file 6: Figure S4 — Four proteins, significant with respect to risk stratification, participate in the statin pathway, which is involved in cholrsterol regulation and lipoprotein remodeling, as it also appeared from the GO analysis. The figure was constructed using WebGestalt web-tool [24]. [file 1756-8722-6-52-S6.pdf]
