# Supplementary material for: Protein biomarkers distinguish between high- and low-risk pediatric acute lymphoblastic leukemia in a tissue specific manner
Source: J Hematol Oncol. 2013 Jul 12;6:52. doi: 10.1186/1756-8722-6-52 (PMC3717072; doi:10.1186/1756-8722-6-52)
Supplement: Additional file 5: Figure S3 — Gene Ontology of common proteins in different samplings. Cholesterol regulation and lipoprotein remodeling appeared to be the most significant biological function in which the proteins participate. The figure was constructed using WebGestalt web-tool [24]. [file 1756-8722-6-52-S5.pdf]

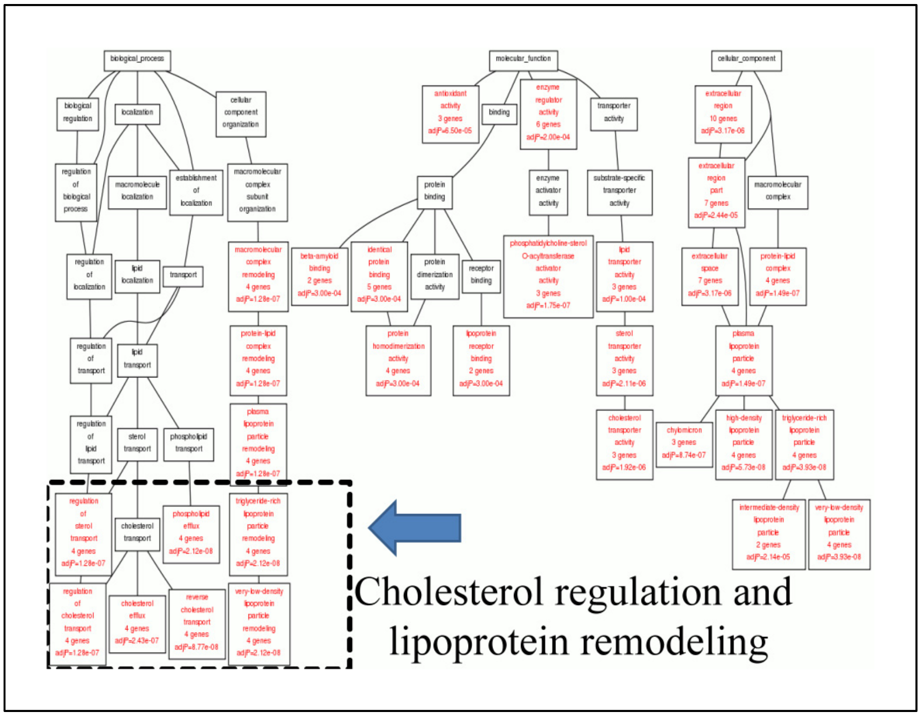

Supplementary Figure 3. Gene Ontology of common proteins in different samplings. Cholesterol regulation and lipoprotein remodeling appeared to be the most significant biological function in which the proteins participate. The figure was constructed using WebGestalt web-tool [24].
